# Supplementary material for: Content-rich biological network constructed by mining PubMed abstracts
Source: BMC Bioinformatics. 2004 Oct 8;5:147. doi: 10.1186/1471-2105-5-147 (PMC528731; doi:10.1186/1471-2105-5-147)
Supplement: Additional File 2 — The original results of the above study (non-essential files are deleted to keep the file size under the limit set by BMC bioinformatics). [file 1471-2105-5-147-S2.bz2 › chilibotAdditionalFile2/dip05/699ID7726836E18027/html/FTSZ_SULA.html]

 


 **FTSZ** and **SULA** 
  
Found 31 abstracts in PubMed, retrieved 05.  
 

 What does Google say? 
 PDF only 
| .edu only 

---

**Interactive relationship** (e.g. stimulation, inhibition, etc)

**Inhibitory relationship**- Instead,  **SulA**  binds the T7 loop surface of  **FtsZ** , opposite the nucleotide binding site, blocking polymer formation.  Ref: 12808143 Proc Natl Acad Sci U S A< MedlineTA>Proc Natl Acad Sci U S A,
- Furthermore, strains carrying various alleles of  **ftsZ** , selected on the basis of resistance to the inhibitor  **SulA** , displayed variable resistance to MinCD.  Ref: 11673433 J Bacteriol, 2001
**Neutral relationship**- Addition of the  **SulA**  protein to the assay leads to rapid disassembly of existing  **FtsZ**  polymers, demonstrating its natural regulatory effect on  **FtsZ**  under the assay conditions.  Ref: 12202250 Anal Biochem, 2002
- **SulA**  halts cell division in Escherichia coli by binding to the major component of the division machinery  **FtsZ** .  Ref: 12808143 Proc Natl Acad Sci U S A< MedlineTA>Proc Natl Acad Sci U S A, 2002

**Non-interactive relationship** (e.g. studied together, co-existance, homology, etc.)

- **SulA**  inhibits cell division neither by binding the nucleotide binding site nor by inducing conformational changes in  **FtsZ** .  Ref: 12808143 Proc Natl Acad Sci U S A< MedlineTA>Proc Natl Acad Sci U S A, 2002
- MinC does not act like  **SulA** , which affects the GTPase activity of  **FtsZ** , and the model can explain how MinC would select for the  **FtsZ**  polymer rather than the monomer.  Ref: 11350934 EMBO J, 2001
- Crystal structure of the SOS cell division inhibitor  [ **SULA** ]   **SulA**  and in complex with  **FtsZ** .  Ref: 12808143 Proc Natl Acad Sci U S A< MedlineTA>Proc Natl Acad Sci U S A, 2001
- We have solved the crystal structure of  **SulA**  alone and in complex with  **FtsZ**  from Pseudomonas aeruginosa.  Ref: 12808143 Proc Natl Acad Sci U S A< MedlineTA>Proc Natl Acad Sci U S A, 2001
